# Effectiveness of regdanvimab treatment for SARS-CoV-2 delta variant, which exhibited decreased *in vitro* activity: a nationwide real-world multicenter cohort study

**DOI:** 10.3389/fcimb.2023.1192512

**Published:** 2023-05-15

**Authors:** Haein Kim, Young Rock Jang, Ji Yeon Lee, Jae-Hoon Ko, Jee Young Lee, Seongcheol Cho, Yong Dae Lee, Junghoon Song, Miri Hyun, Hyun Ah Kim, Soyoon Hwang, Sangmi Ryou, Yoo Jin Na, Joo-Yeon Lee, Changhee Lee, Nan Young Lee, Seunghwan Shin, Ki Tae Kwon, Jin Yong Kim, Kyong Ran Peck

**Affiliations:** ^1^ Division of Infectious Diseases, Department of Medicine, Samsung Medical Center, Sungkyunkwan University School of Medicine, Seoul, Republic of Korea; ^2^ Division of Infectious Diseases, Department of Internal Medicine, Incheon Medical Center, Incheon, Republic of Korea; ^3^ Division of Infectious Diseases, Department of Internal Medicine, Keimyung University Dongsan Hospital, Keimyung University School of Medicine, Daegu, Republic of Korea; ^4^ Department of Internal Medicine, Seoul Red Cross Hospital, Seoul, Republic of Korea; ^5^ Division of Infectious Diseases, Department of Internal Medicine, Kyungpook National University Chilgok Hospital, School of Medicine, Kyungpook National University, Daegu, Republic of Korea; ^6^ Center for Emerging Virus Research, Korea National Institute of Health, Korea Disease Control and Prevention Agency, Cheongju, Republic of Korea; ^7^ College of Veterinary Medicine and Virus Vaccine Research Center, Gyeongsang National University, Jinju, Republic of Korea; ^8^ Department of Clinical Pathology, School of Medicine, Kyungpook National University, Daegu, Republic of Korea

**Keywords:** SARS-CoV-2, delta variant, regdanvimab, monoclonal antibody, outcome

## Abstract

**Background:**

Immune-evading severe acute respiratory syndrome coronavirus 2 (SARS-CoV-2) variants are emerging continuously. The clinical effectiveness of monoclonal antibody agents that exhibit decreased *in vitro* activity against SARS-CoV-2 variants needs to be elucidated.

**Methods:**

A nationwide, multicenter, retrospective cohort study was designed to evaluate the effectiveness of regdanvimab, an anti-SARS-CoV-2 monoclonal antibody agent. Regdanvimab was prescribed in South Korea before and after the emergence of the delta variant, against which the *in vitro* activity of regdanvimab was decreased but present. Mild to moderate coronavirus 2019 (COVID-19) patients with risk factors for disease progression who were admitted within seven days of symptom onset were screened in four designated hospitals between December 2020 and September 2021. The primary outcomes, O_2_ requirements and progression to severe disease within 21 days of admission, were compared between the regdanvimab and supportive care groups, with a subgroup analysis of delta variant–confirmed patients.

**Results:**

A total of 2,214 mild to moderate COVID-19 patients were included, of whom 1,095 (49.5%) received regdanvimab treatment. In the analysis of the total cohort, significantly fewer patients in the regdanvimab group than the supportive care group required O_2_ support (18.4% vs. 27.1%, *P* < 0.001) and progressed to severe disease (4.0% vs. 8.0%, *P* < 0.001). In the multivariable analysis, regdanvimab was significantly associated with a decreased risk for O_2_ support (HR 0.677, 95% CI 0.561–0.816) and progression to severe disease (HR 0.489, 95% CI 0.337–0.709). Among the 939 delta-confirmed patients, O_2_ support (21.5% vs. 23.5%, *P* = 0.526) and progression to severe disease (4.2% vs. 7.3%, *P* = 0.055) did not differ significantly between the regdanvimab and supportive care groups. In the multivariable analyses, regdanvimab treatment was not significantly associated with a decreased risk for O_2_ support (HR 0.963, 95% CI 0.697–1.329) or progression to severe disease (HR 0.665, 95% CI 0.349–1.268) in delta-confirmed group.

**Conclusions:**

Regdanvimab treatment effectively reduced progression to severe disease in the overall study population, but did not show significant effectiveness in the delta-confirmed patients. The effectiveness of dose increment of monoclonal antibody agents should be evaluated for variant strains exhibiting reduced susceptibility.

## Introduction

Immune-evading severe acute respiratory syndrome coronavirus 2 (SARS-CoV-2) variants are emerging continuously. On March 11, 2021, the delta variant (B.1.617.2) became the fourth variant of concern (VOC), and it dominated the global SARS-CoV-2 outbreak during the second half of 2021 (WHO, 2023a). The omicron variant (B.1.1.529) became the fifth VOC on November 26, 2021, and its subvariants have been the dominant variants circulating globally since then, accounting for > 98% of viral sequences since February 2022 (WHO, 2023b). Anti-SARS-CoV-2 monoclonal antibody agents (mAbs) effectively reduce the progression of coronavirus disease 2019 (COVID-19) (Dougan et al., 2021; Weinreich et al., 2021; Gupta et al., 2022; Yamasoba et al., 2022), but their activities are easily affected by emerging mutations (VanBlargan et al., 2022). Four anti-SARS-CoV-2 mAbs, bamlanivimab/etesevimab (Eli Lilly and Company, IN, USA), casirivimab/imdevimab (Ronapreve; Regeneron Pharmaceuticals Inc., NY, USA), sotrovimab (Xevudy; GlaxoSmithKline LLC, NC, USA), and bebtelovimab (Eli Lilly and Company, IN, USA), received Emergency Use Authorizations (EUAs) from the Food and Drug Administration (FDA) (NIH, 2022), but they are not currently in use due to the loss of *in vitro* activity against omicron subvariants (Planas et al., 2021; Takashita et al., 2022). However, the clinical effectiveness of mAbs against variants that exhibit reduced *in vitro* susceptibility has not been elucidated.

Regdanvimab (Regkirona; Celltrion Inc., Incheon, South Korea), an anti-SARS-CoV-2 mAb, received approval in South Korea in February 2021 for the treatment of mild to moderate COVID-19 patients with risk factors for progression to severe disease (Kim et al., 2021; Syed, 2021), and it has also been approved for use in Europe and Australia (Syed, 2021; Nham et al., 2022a). In a phase 2/3 randomized controlled trial, regdanvimab shortened the duration of viable SARS-CoV-2 shedding and reduced hospitalization and O_2_ therapy in patients with mild to moderate COVID-19 (Streinu-Cercel et al., 2022). In the real world, several studies have shown that regdanvimab treatment was associated with a decreased risk of progression to severe disease in the pre-delta period (Lee et al., 2021; Streinu-Cercel et al., 2022). However, few data are available on the clinical effectiveness of regdanvimab treatment for the delta variant, although *in vitro* and animal experiments suggest that its activity is decreased but still effective (Lee et al., 2022). Previous studies have shown that regdanvimab treatment tended to reduce disease progression in delta variant infections, but that effect did not reach statistical significance due to the small number of patients and insufficient statistical power of those investigations (Ryu et al., 2021; Lee et al., 2022; Jang et al., 2023). Although regdanvimab is currently not in use because of its reduced activity against omicron variants, it remains a crucial question whether it was clinically effective for the delta variant, which exhibited decreased but still present susceptibility to regdanvimab (VanBlargan et al., 2022). To answer that question, we conducted a nationwide, multicenter, retrospective cohort study evaluating the effectiveness of regdanvimab treatment in patients infected with ancestral strains and the delta variant.

## Methods

### Study population and design

A retrospective observational cohort study was conducted to evaluate the effectiveness of regdanvimab in moderating the clinical outcomes of mild to moderate COVID-19 patients who were admitted to four COVID-19-designated hospitals in South Korea between December 2020 and September 2021. The study period includes the pre-delta period and the delta-outbreak period in South Korea. Domestic cases of the delta variant were detected beginning in the 2^nd^ week of May 2021 and increased from June 2021. Because the total number of confirmed domestic cases of the delta variant in South Korea was limited to nine in May 2021, we defined the pre-delta period as December 2020 to May 2021 for this study (KDCA, 2021; KDCA, 2022a), and we defined the delta-outbreak period from June 2021 to September 2021. Because the proportion of the delta variant increased gradually during this period (KDCA, 2022a), we included only delta-confirmed cases. Delta variant infections were confirmed by double-multiplex real-time reverse transcription polymerase chain reaction (RT-PCR) using a PowerChek SARS-CoV-2 S-gene mutation detection kit (Kogene Biotech Co Ltd, Seoul, South Korea), spike protein sequencing, or whole genome sequencing (Il-Hwan Kim et al., 2021; Park et al., 2022). Delta variants were identified based on the P681R and L452R mutations.

During the whole study period, mild to moderate COVID-19 patients at risk of disease progression were hospitalized at general COVID-19-designated hospitals, and worsening COVID-19 patients with O_2_ requirements of more than 5 L per min *via* nasal prong or facial mask were referred to tertiary care hospitals (Lee et al., 2021). Patients with the following inclusion criteria, adopted from the regdanvimab treatment indications, were included: (1) confirmed SARS-CoV-2 infection, (2) symptom onset within seven days before admission, (3) no oxygen requirement (SpO_2_ > 94% in room air), and (4) high risk for disease progression (age ≥ 50 years plus one of the following underlying diseases, diabetes mellitus, cardiovascular disease, chronic renal disease, or chronic lung disease, and body mass index (BMI) ≥ 30 kg/m^2^; or age ≥ 60 years with or without underlying disease). Confirmation of SARS-CoV-2 infection was performed using RT-PCR assay test kits approved by the Korean Ministry of Food and Drug Safety (Joo et al., 2021). Because the present study is retrospective, attending physicians at the four participating hospitals prescribed regdanvimab to willing, indicated patients at their own discretion after the drug became available in February 2021. COVID-19 patients admitted from February to September 2021 were thus likely to receive regdanvimab treatment if indicated, whereas those admitted from December 2020 to February 2021 did not receive the drug, with an overlap period in February 2021. The enrolled patients were classified into the regdanvimab group or supportive care group, with patients in the regdanvimab group receiving a dose of 40 mg/kg intravenously. The enrolled patients were followed until their day of discharge or referral. The primary outcomes were O_2_ requirement and progression to severe disease within 21 days of admission, and secondary outcomes were the requirement for other treatment modalities (remdesivir, corticosteroid, or antibiotics), duration of hospital stay, and all-cause mortality during hospitalization. Some of the clinical data for the present cohort, mainly those from the pre-delta period, were published previously (Lee et al., 2021; Ryu et al., 2021; Streinu-Cercel et al., 2022). Review by the Institutional Review Board was exempted because this investigation was conducted as part of a public health response, and minimal risk was expected to the participating patients.

### Data collection

We reviewed information about baseline characteristics: age, gender, date of symptom onset/diagnosis/admission, underlying diseases, COVID-19 vaccination history, patient management, and clinical outcomes. Clinical status at admission was evaluated using SpO_2_, radiologic evidence for pneumonia, complete blood count, chemistry profile, and C-reactive protein (CRP) levels. The primary outcomes were assessed based on ordinal disease severity scores (**Supplementary Materials**) (Sung et al., 2020). O_2_ support *via* nasal prong was the same as a severity score of 3, and the composite outcome indicating progression to severe disease was defined as progression to a severity score of 4 to 8, including referral to a tertiary care hospital due to increasing O_2_ requirements (Lee et al., 2021). During the study period, two adenoviral vector vaccines (ChAdOx1 nCoV-19, Oxford-AstraZeneca, and Ad26.COV2.S, Janssen) and two mRNA vaccines (BNT162b2, Pfizer-BioNTech, and mRNA-1273, Moderna) were introduced in South Korea, and heterologous vaccination was allowed (Bae et al., 2022; Nham et al., 2022b). A single dose of the Ad26.COV2.S vaccine or two doses of the other vaccines was defined as complete vaccination. Receiving only one dose of the ChAdOx1 nCoV-19, BNT162b2, or mRNA-1273 vaccine was classified as incomplete vaccination.

### Statistical analyses

We analyzed categorical variables using *chi* square or Fisher exact tests, and continuous variables were analyzed using the Student *t*-test or Mann-Whitney *U* test. The 21-day probability of disease progression was described by the Kaplan-Meier method and compared using the log-rank test. Potential risk factors for disease progression within 21 days were evaluated using Cox proportional hazard models. Univariable analyses of factors influencing the outcomes were performed, and variables with *P* < 0.1 were included in the multivariable analyses of regdanvimab treatment. When a continuous variable was statistically significant in the univariable analysis, it was converted into a categorical variable using interquartile ranges, the receiver operating characteristic curve, or known normal limits, and the variable with the highest hazard ratio was included in the multivariable analyses. All tests of significance were two-tailed, and a *P* value < 0.05 was considered statistically significant. All statistical analyses were performed using SPSS^®^, version 27.0 K for Windows (SPSS Inc, Chicago, IL, USA), and GraphPad Prism 9.0 (GraphPad Software, San Diego, CA, USA) was used to develop the figures.

## Results

### Baseline characteristics of mild to moderate COVID-19 patients, total cohort

**Figure 1:**
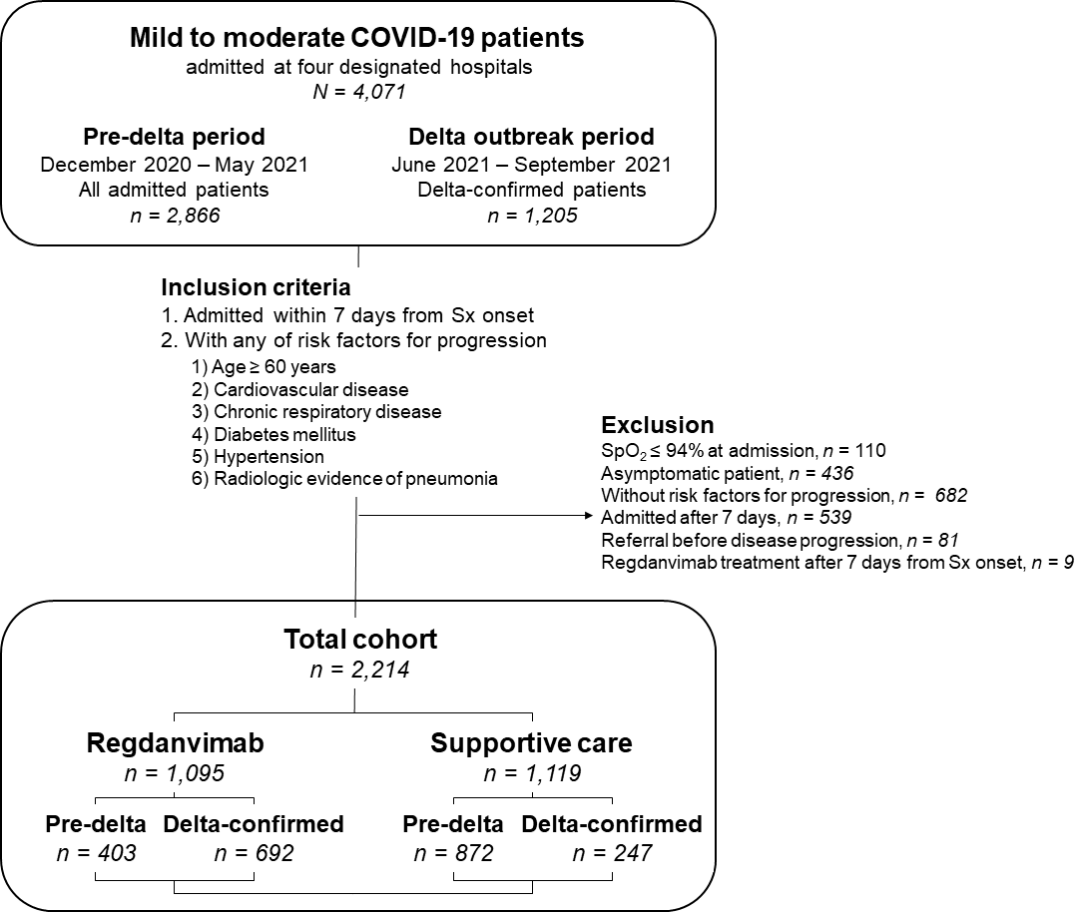
Schematic view of the study population. Mild to moderate COVID-19 patients admitted at four COVID-19-designated hospitals in South Korea between December 2020 and September 2021 were screened. During the pre-delta period (December 2020 to May 2021), all admitted patients were enrolled, whereas only delta-confirmed patients were included during the delta outbreak period (June 2021 to September 2021). Abbreviations: COVID-19, coronavirus disease 2019; Sx, symptom; SpO_2_, saturation of peripheral oxygen.

During the study period, 4,071 mildly ill COVID-19 patients were admitted to the four general hospitals. After excluding 1,857 patients, we included 2,214 patients with risk factors for progression to severe disease who were admitted within seven days of symptom onset in this study (**Figure 1**). The patients were divided into the regdanvimab group (n = 1,095; 403 pre-delta and 692 delta-confirmed patients) and the supportive care group (n = 1,119; 872 pre-delta and 247 delta-confirmed patients). The baseline characteristics of all 2,214 mild to moderate COVID-19 patients are presented in **Table 1**. Patients in the regdanvimab group were generally younger, admitted earlier, and had a higher BMI, complete vaccination, and a SARS-CoV-2 viral load than those in the supportive care group (*P* < 0.001 in all comparisons). In addition, they were significantly more likely to have pneumonia (*P* < 0.001) than patients in the supportive care group. The average values of the initial laboratory tests were all within normal ranges except for CRP (2.0 ± 3.0 mg/dL) and lactate dehydrogenase (LDH, 311.9 ± 134.7). The CRP level was significantly higher in the regdanvimab group (2.2 ± 2.9 mg/dL) than the supportive care group (1.9 ± 3.2 mg/dL, *P* = 0.034), and the LDH level was significantly lower in the regdanvimab group (300.6 ± 122.0 IU/L) than the supportive care group (322.8 ± 145.2 IU/L, *P* < 0.001). Patients in the regdanvimab group were significantly less likely to have respiratory disease (*P* = 0.009), diabetes mellitus (*P* = 0.013), and hypertension (*P* = 0.037).

**Table 1:** Baseline characteristics of mild to moderate COVID-19 patients, total cohort.

Characteristic	Regdanvimab (n = 1,095)	Supportive care (n = 1,119)	*P* value
Demographics			
Age, years	53 (41–65)	59 (45–69)	<0.001
Male gender	549 (50.1)	544 (48.6)	0.474
BMI	25.1 ± 4.0	24.7 ± 4.0	0.011
BMI ≥ 25 kg/m^2^	513 (46.8)	482 (43.1)	0.074
BMI ≥ 30 kg/m^2^	104 (9.5)	96 (8.6)	0.451
Symptom onset to admission, days	2 (1–4)	3 (1–5)	<0.001
Vaccination (incomplete or complete)	229 (20.9)	77 (6.9)	<0.001
Vaccination (complete only)	81 (7.4)	31 (2.8)	<0.001
Initial presentation			
Initial Ct value (NP swab, RdRp)	18.2 ± 5.8	20.0 ± 6.0	<0.001
SpO_2_, %	97.3 ± 1.0	97.5 ± 1.1	0.005
SpO_2_ < 97%	203 (18.5)	203 (18.1)	0.809
Pneumonia	906 (82.7)	818 (73.1)	<0.001
Initial laboratory tests			
WBC count, x10^3^/μL	4.8 ± 1.8	5.1 ± 2.5	0.005
Lymphocyte count, x10^3^/μL	1.2 ± 5.3	1.3 ± 6.7	<0.001
Platelet count, x10^3^/μL	187.1 ± 65.0	192.3 ± 70.7	0.072
Total bilirubin, mg/dL	0.6 ± 0.5	0.6 ± 0.3	0.634
Albumin, g/dL	4.42 ± 0.36	4.37 ± 0.39	0.002
AST, IU/L	34.9 ± 22.7	35.1 ± 37.3	0.867
ALT, IU/L	33.0 ± 28.2	33.6 ± 42.2	0.712
BUN, mg/dL	14.7 ± 22.2	14.8 ± 9.2	0.824
Creatinine, mg/dL	0.9 ± 0.4	0.9 ± 0.7	0.128
LDH, IU/L	300.6 ± 122.0	322.8 ± 145.2	<0.001
CRP, mg/dL	2.2 ± 2.9	1.9 ± 3.2	0.034
Underlying diseases			
Cardiovascular disease	73 (6.7)	83 (7.4)	0.490
Respiratory disease	32 (2.9)	57 (5.1)	0.009
Diabetes mellitus	158 (14.4)	205 (18.3)	0.013
Hypertension	339 (31.0)	393 (35.1)	0.037
Liver disease	2 (0.2)	3 (0.3)	1.000
Renal disease	4 (0.4)	8 (0.7)	0.263
Solid cancer, curative	32 (2.9)	40 (3.6)	0.387
Solid cancer, metastasis	8 (0.7)	11 (1.0)	0.520
Solid organ transplant	1 (0.1)	0 (0.0)	0.312
SARS-CoV-2 virus subtype			
Pre-delta	403 (36.8)	872 (77.9)	<0.001
Delta-confirmed	692 (63.2)	247 (22.1)	<0.001

Data are expressed as the number (%) of patients, mean ± SD, or median (IQR) unless indicated otherwise.

COVID-19, coronavirus disease 2019, BMI, body mass index; Ct, cycle threshold; NP, nasopharyngeal; RdRp, RNA-dependent RNA polymerase; SpO_2_, saturation of percutaneous oxygen; WBC, white blood cell; AST, aspartate aminotransferase; ALT, alanine aminotransferase; BUN, blood urea nitrogen; CPK, creatine phosphokinase; LDH, lactate dehydrogenase; CRP, C-reactive protein; SARS-CoV-2, severe acute respiratory syndrome coronavirus 2; SD, standard deviation; IQR, interquartile range

### Treatment and outcomes of the total cohort

**Figure 2:**
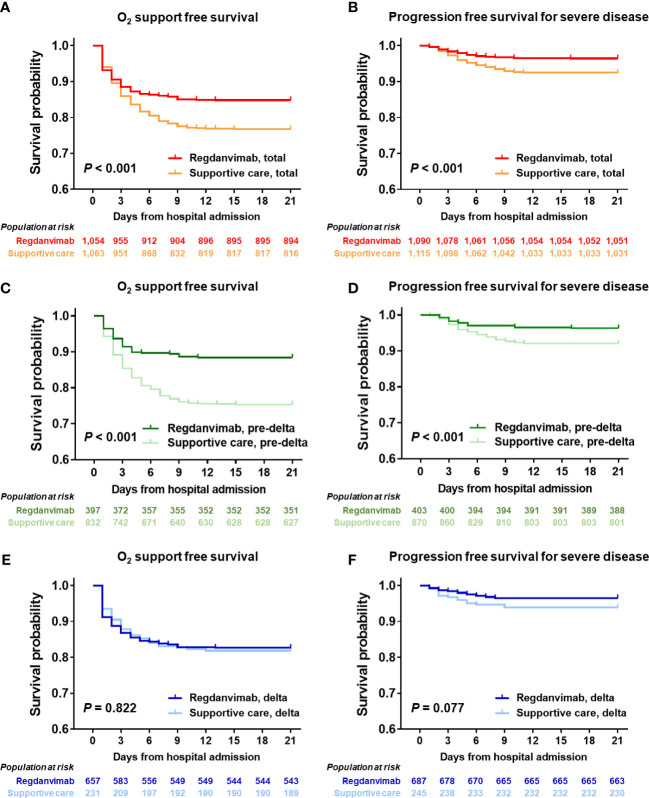
Progression-free survival analysis in the total cohort and delta-confirmed subgroup. The 21-day probabilities for O_2_ support–free survival **(A)** and progression free survival for severe disease **(B)** were evaluated in the total cohort, and the regdanvimab group showed clinical benefit for both outcomes. Significant benefits were found in the pre-delta subgroup for O_2_ support–free survival **(C)** or progression free survival for severe disease **(D)**, whereas those were not found in the delta-confirmed subgroup for O_2_ support–free survival **(E)** or progression free survival for severe disease **(F)**.

We compared the treatment and clinical outcomes of the regdanvimab and supportive care groups (**Table 2**). Patients in the regdanvimab group received regdanvimab treatment an average of 3.6 days after symptom onset and 1.1 days after admission. 279 patients (13%) received remdesivir, without a significant difference between the groups. Patients in the regdanvimab group were less likely to receive steroid (16.7% vs. 22.1%, *P* = 0.001) or antibiotic treatment (4.6% vs. 6.6%, *P* = 0.036) than those in the supportive care group. After admission, significantly fewer patients in the regdanvimab group required O_2_ supplementation *via* nasal prong (18.4% vs. 27.1%, *P* < 0.001) and progressed to severe disease (4.0% vs. 8.0%, *P* < 0.001), compared with those in the supportive care group. When 21-day O_2_ support–free survival was calculated using the Kaplan-Meier method, significantly fewer patients in the regdanvimab group required O_2_ support than in the supportive care group (*P* < 0.001, **Figure 2A**). The 21-day progression-free survival for severe disease was also significantly better in patients in the regdanvimab group than the supportive care group (*P* < 0.001, **Figure 2B**). Significantly more patients in the regdanvimab group than the supportive care group were discharged after recovery without referral to tertiary care centers (98.2% vs. 94.5%, *P* < 0.001). The hospital stays were also shorter in the regdanvimab group (10.3 ± 3.7 days) than the supportive care group (12.3 ± 12.5 days, *P* < 0.001).

**Table 2:** Treatment and outcomes of the regdanvimab and supportive care groups, total cohort.

Variables	Regdanvimab (n = 1,095)	Supportive care (n = 1,119)	*P* value
Regdanvimab			
Regdanvimab treatment	1,095 (100)	0 (0.0)	NA
Interval from symptom onset to regdanvimab, days	3.6 ± 2.0	NA	NA
Interval from admission to regdanvimab, days	1.1 ± 1.3	NA	NA
Remdesivir, steroid, and antibiotics			
Remdesivir treatment	128 (11.7)	151 (13.5)	0.201
Interval from admission to remdesivir, days	2.5 ± 2.1	3.3 ± 3.1	0.006
Steroid treatment	183 (16.7)	247 (22.1)	0.001
Interval from admission to steroid, days	2.5 ± 2.7	3.1 ± 3.1	0.042
Antibiotic treatment	50 (4.6)	74 (6.6)	0.036
Interval from admission to antibiotics, days	1.7 ± 2.1	4.2 ± 3.7	<0.001
Outcome measures			
O_2_ supplementation *via* nasal prong	201 (18.4)	303 (27.1)	<0.001
Interval from admission to nasal prong, days	17.5 ± 7.4	16.1 ± 8.2	<0.001
Composite outcome for progression to severe disease	44 (4.0)	90 (8.0)	<0.001
Interval from admission to composite outcome, days	20.3 ± 3.4	19.7 ± 4.5	<0.001
O_2_ supplement *via* facial mask	10 (0.9)	34 (3.0)	<0.001
Interval from admission to facial mask, days	4 (3–5)	5 (3–7)	0.002
O_2_ supplement *via* HFNC	26 (2.4)	40 (3.6)	0.097
Interval from admission to HFNC, days	3 (1–5)	5 (2–7)	<0.001
Referral to tertiary care center	20 (1.8)	57 (5.1)	<0.001
Interval from admission to referral, days	4 (2–6)	5 (3–8)	0.001
**Live discharge after recovery without referral**	1075 (98.2)	1058 (94.5)	<0.001
Interval from admission to discharge, days	10.3 ± 3.7	12.3 ± 12.5	<0.001
**In-hospital mortality during follow-up period**	0 (0.0)	4 (0.4)	0.125

Data are expressed as the number (%) of patients, mean ± SD, or median (IQR) unless indicated otherwise.

NA, not applicable; HFNC, high flow nasal cannula; SD, standard deviation; IQR, interquartile range.

To identify potential confounding factors for 21-day disease progression probability, we considered variables of clinical importance in the univariable analyses and then included those with *P* values < 0.1 in the multivariable analysis (**Table 3** and **Supplementary Tables 1, 2**). The independent risk factors associated with O_2_ support *via* nasal prong were found to be age ≥ 70 years (hazard ratio (HR) 2.037, 95% confidence interval (CI) 1.643–2.526), BMI ≥ 25 kg/m^2^ (HR 1.504, 95% CI 1.254–1.803), days from symptom onset (HR 1.097, 95% CI 1.048–1.148), SpO_2_ < 97% (HR 1.669, 95% CI 1.371–2.033), pneumonia (HR 1.594, 95% CI 1.246–2.039), neutrophilia > 3500/μL (HR 1.390, 95% CI 1.157–1.670), thrombocytopenia < 150 x 10^3^/μL (HR 1.830, 95% CI 1.518–2.206), albumin < 4.0 g/dL (HR 1.376, 95% CI 1.090–1.736), creatinine elevation > 1.2 mg/dL (HR 1.318, 95% CI 1.007–1.725), CRP elevation > 1.5 mg/dL (HR 2.487, 95% CI 2.027–3.051), diabetes mellitus (HR 1.278, 95% CI 1.026–1.593), and metastatic solid cancer (HR 3.741, 95% CI 1.907–7.339). Regdanvimab treatment (HR 0.677, 95% CI 0.561–0.816) and complete vaccination (HR 0.374, 95% CI 0.212–0.660) were associated with a reduced risk for O_2_ support *via* nasal prong. The independent risk factors associated with progression to severe disease were age > 70 years (HR 1.574, 95% CI 1.039–2.383), male gender (HR 1.480, 95% CI 1.027–2.135), BMI ≥ 25 kg/m^2^ (HR 1.830, 95% CI 1.283–2.610), SpO_2_ < 97% (HR 3.104, 95% CI 2.189–4.402), pneumonia (HR 1.696, 95% CI 1.043–2.759), lymphopenia < 1500/μL (HR 2.182, 95% CI 1.291–3.687), thrombocytopenia (HR 1.501, 95% CI 1.051–2.142), albumin < 4.0 g/dL (HR 2.134, 95% CI 1.407–3.236), and CRP elevation > 5 mg/dL (HR 2.860, 95% CI 1.956–4.182). Regdanvimab treatment (HR 0.489, 95% CI 0.337–0.709) was also significantly associated with a reduced risk for progression to severe disease.

**Table 3:** Multivariable analysis of 21-day disease progression probability, total cohort.

Factors for disease progression	O_2_ support		Progression to severe disease	
	HR (95% CI)	*P* value	HR (95% CI)	*P* value
Age > 70 years	2.037 (1.643–2.526)	<0.001	1.574 (1.039–2.383)	0.032
Male gender	0.902 (0.748–1.086)	0.277	1.480 (1.027–2.135)	0.036
BMI ≥ 25 kg/m^2^	1.504 (1.254–1.803)	<0.001	1.830 (1.283–2.610)	0.001
Symptom onset to admission, days	1.097 (1.048–1.148)	<0.001	0.985 (0.898–1.081)	0.750
Vaccination (complete only)	0.374 (0.212–0.660)	0.001	0.180 (0.025–1.311)	0.091
SpO_2_ < 97%	1.669 (1.371–2.033)	<0.001	3.104 (2.189–4.402)	<0.001
Pneumonia	1.594 (1.246–2.039)	<0.001	1.696 (1.043–2.759)	0.033
Neutrophil > 3500/μL	1.390 (1.157–1.670)	<0.001		
Lymphopenia (< 1500/μL)			2.182 (1.291–3.687)	0.004
Thrombocytopenia (< 150x10^3^/μL)	1.830 (1.518–2.206)	<0.001	1.501 (1.051–2.142)	0.026
Albumin <4.0 g/dL	1.376 (1.090–1.736)	0.007	2.134 (1.407–3.236)	<0.001
BUN elevation (> 19 mg/dL)			1.441 (0.930–2.233)	0.102
Creatinine elevation (> 1.2 mg/dL)	1.318 (1.007–1.725)	0.044		
LDH elevation (> 400 IU/L)			1.189 (0.804–1.759)	0.386
CRP elevation (> 1.5 mg/dL)	2.487 (2.027–3.051)	<0.001		
CRP elevation (> 5 mg/dL)			2.860 (1.956–4.182)	<0.001
Diabetes mellitus	1.278 (1.026–1.593)	0.029		
Hypertension	1.137 (0.928–1.394)	0.216		
Renal disease	0.756 (0.323–1.770)	0.519		
Solid cancer, metastasis	3.741 (1.907–7.339)	<0.001		
**Delta-confirmed**			1.045 (0.682–1.601)	0.839
**Regdanvimab treatment**	0.677 (0.561–0.816)	<0.001	0.489 (0.337–0.709)	<0.001

HR, hazard ratio; CI, confidence interval; BMI, body mass index; SpO_2_, saturation of percutaneous oxygen; WBC, white blood cell; ALT, alanine aminotransferase; AST, aspartate aminotransferase; BMI, body mass index; BUN, blood urea nitrogen; LDH, lactate dehydrogenase; CRP, C-reactive protein.

### Comparison of the pre-delta and delta-confirmed patients

Among the 2,214 mild to moderate COVID-19 patients, 939 (42.4%) were infected with the delta variant. The delta-confirmed patients were generally younger (*P* < 0.001), admitted later (*P* < 0.001), had a higher BMI (*P* = 0.003), and had a higher SARS-CoV-2 viral load (*P* < 0.001), compared with patients from the pre-delta period (**Supplementary Table 3**). One-third of the delta-confirmed patients had received at least one vaccine dose, and 10% of them were completely vaccinated, whereas none of the patients in the pre-delta period were vaccinated (*P* < 0.001). In addition, the delta-confirmed patients were significantly more likely than patients from the pre-delta period to have pneumonia (*P* < 0.001) or an elevated CRP level (*P* < 0.001). The delta-confirmed patients were significantly less likely to have diabetes mellitus (*P* < 0.001), hypertension (*P* < 0.001), and metastatic solid cancer (*P* = 0.001) than those in the pre-delta period.

When we compared the treatment and clinical outcomes between the pre-delta and delta-confirmed patients (**Supplementary Table 4**), we found that 692 (73.7%) of 939 delta-confirmed patients received regdanvimab treatment, which was a higher proportion than in the pre-delta period (31.6%, *P* < 0.001). Significantly more delta-confirmed patients required remdesivir (15.2% vs. 10.7%, *P* = 0.001) and steroid treatment (26.0% vs. 14.6%, *P* < 0.001) than in the pre-delta period. However, O_2_ supplementation *via* nasal prong (22.0% vs. 23.3%, *P* = 0.488) and progression to severe disease (5.0% vs. 6.8%, *P* = 0.076) did not differ significantly between the groups. In addition, significantly fewer delta-confirmed patients were referred to a tertiary care center, compared with those in the pre-delta cohort (2.2% vs. 4.4%, *P* = 0.006). Likewise, significantly more delta-confirmed patients were discharged after recovery without referral to a tertiary care center (97.8% vs. 95.3%, *P* = 0.002), compared with those in the pre-delta cohort. The hospital stays of delta-confirmed patients (9.8 ± 3.5 days) were also shorter than those of the pre-delta cohort (12.4 ± 11.7 days, *P* < 0.001).

### Baseline characteristics, treatment, and outcomes of the delta-confirmed subgroup

The baseline characteristics of the study population infected with the delta variant are presented in **Supplementary Table 5**. The delta-confirmed patients in the regdanvimab treatment group were generally older (*P* < 0.001), admitted earlier (*P* = 0.002), had a higher BMI ≥ 25 kg/m^2^ (*P* = 0.031), pneumonia (*P* = 0.017), higher CRP level (*P* = 0.049), and lower LDH level (*P* = 0.001) than those in the delta-confirmed subgroup who received supportive care. The treatment and outcomes of the delta-confirmed patients are presented in **Supplementary Table 6**. The percentages who received remdesivir and antibiotic treatment did not differ between the groups, but the steroid requirement was significantly lower in patients treated with regdanvimab than in those who received supportive care (21.0% vs. 40.1%, *P* < 0.001). In the delta-confirmed subgroup, O_2_ supplementation *via* nasal prong, progression to severe disease, and discharge after recovery without referral to a tertiary care center did not differ significantly between the regdanvimab and supportive care groups. In the survival analysis using the Kaplan-Meier method, neither O_2_ support–free survival (*P* = 0.822, **Figure 2E**) nor progression free survival for severe disease (*P* = 0.077, **Figure 2F**) differed between the regdanvimab and supportive care groups in the delta-confirmed subgroup, whereas significantly fewer patients in the regdanvimab group required O_2_ support and progressed to severe disease than in the supportive care group in the pre-delta period (both *P* < 0.001, **Figures 2C, D**).

To adjust for potential confounding factors in the 21-day disease progression probability of the delta-confirmed patients, we considered variables of clinical importance in a univariable analysis and then included those with *P* values < 0.1 in a multivariable analysis (**Table 4** and **Supplementary Tables 7, 8**). Among the variables found to be significant in the univariable analyses, BMI ≥ 30 kg/m^2^ (HR 2.301, 95% CI 1.614–3.283), days from symptom onset (HR 1.159, 95% CI 1.075–1.249), SpO_2_ < 97% (HR 1.861, 95% CI 1.355–2.557), lymphopenia < 1500/μL (HR 1.709, 95% CI 1.131–2.583), thrombocytopenia < 150 x 10^3^/μL (HR 1.742, 95% CI 1.300–2.335), albumin < 4.0 g/dL (HR 1.871, 95% CI 1.292–2.708), LDH level (HR 0.999, 95% CI 0.998–1.000), and CRP elevation > 1.5 mg/dL (HR 3.021, 95% CI 2.141–4.261) remained significant risk factors for O_2_ supplementation *via* nasal prong in the multivariable analysis. The independent risk factors associated with progression to severe disease in the delta-confirmed subgroup were BMI ≥ 25 kg/m^2^ (HR 1.987, 95% CI 1.068–3.697), SpO_2_ < 97% (HR 4.309, 95% CI 2.411–7.701), lymphopenia < 1500/μL (HR 5.119, 95% CI 1.233–21.259), albumin < 4.0 g/dL (HR 3.382, 95% CI 1.790–6.389), and CRP elevation > 1.5 mg/dL (HR 4.169, 95% CI 1.841–9.442). The initial Ct value (HR 0.934, 95% CI 0.880–0.992), which would be associated with a low viral load, was significantly associated with a reduced risk for progression to severe disease. However, regdanvimab treatment was not associated with reduced risk for either O_2_ supplementation *via* nasal prong or progression to severe disease in the multivariable analyses (*P* = 0.817 and *P* = 0.216, respectively), in contrast to the total cohort.

**Table 4:** Multivariable analysis of 21-day disease progression probability, delta-confirmed subgroup.

Factors for disease progression	O_2_ support		Progression to severe disease	
	HR (95% CI)	*P* value	HR (95% CI)	*P* value
Age > 60 years	0.966 (0.642–1.452)	0.867		
BMI ≥ 25 kg/m^2^			1.987 (1.068–3.697)	0.030
BMI ≥ 30 kg/m^2^	2.301 (1.614–3.283)	<0.001		
Symptom onset to admission, days	1.159 (1.075–1.249)	<0.001	1.001 (0.848–1.182)	0.992
Vaccination (complete only)	0.602 (0.313–1.156)	0.127	0.231 (0.032–1.691)	0.149
SpO_2_ < 97%	1.861 (1.355–2.557)	<0.001	4.309 (2.411–7.701)	<0.001
Pneumonia	1.403 (0.648–3.039)	0.391		
Initial Ct value (NP swab, RdRp)	0.980 (0.954–1.006)	0.133	0.934 (0.880–0.992)	0.025
Lymphopenia (< 1500/μL)	1.709 (1.131–2.583)	0.011	5.119 (1.233–21.259)	0.025
Thrombocytopenia (< 150x10^3^/μL)	1.742 (1.300–2.335)	<0.001	1.144 (0.633–2.069)	0.656
Albumin < 4.0 g/dL	1.871 (1.292–2.708)	0.001	3.382 (1.790–6.389)	<0.001
BUN elevation (> 19 mg/dL)	1.349 (0.854–2.129)	0.199	1.929 (0.873–4.261)	0.104
LDH, IU/L	0.999 (0.998–1.000)	0.008		
LDH elevation (> 400 IU/L)			1.098 (0.566–2.129)	0.782
CRP elevation (> 1.5 mg/dL)	3.021 (2.141–4.261)	<0.001	4.169 (1.841–9.442)	0.001
Diabetes mellitus	1.238 (0.804–1.908)	0.333		
**Regdanvimab treatment**	0.963 (0.697–1.329)	0.817	0.665 (0.349–1.268)	0.216

HR, hazard ratio; CI, confidence interval; BMI, body mass index; SpO_2_, saturation of percutaneous oxygen; BUN, blood urea nitrogen; LDH, lactate dehydrogenase; CRP, C-reactive protein.

## Discussion

Anti-SARS-CoV-2 mAbs targeting the spike protein have been shown to have clinical advantages by providing passive immunization, but the clinical activity of each anti-SARS-CoV-2 mAb against circulating SARS-CoV-2 variants differs depending on mutations in the spike proteins.

Bamlanivimab/etesevimab, casirivimab/imdevimab, and sotrovimab retained their *in vitro* activity against the delta variant and showed clinical effectiveness during the delta-dominated outbreak period (Bierle et al., 2021; Gottlieb et al., 2021; Gupta et al., 2022). However, most mAb agents exhibited markedly reduced activity against the omicron variant and its subvariants, and the current guidelines do not recommend the use of mAb agents to treat those variants (NIH, 2022). Regdanvimab received an EUA from the Korea Ministry of Food and Drug Safety on February 5, 2021, and was widely used during the pre-delta and delta variant periods in South Korea. Previous studies conducted during the pre-delta period showed that regdanvimab treatment effectively reduced the progression to severe disease in mild to moderate COVID-19 patients (Lee et al., 2021; Jang et al., 2023), but real-world data for the delta variant are few (Ryu et al., 2021; Jang et al., 2023). The delta variant exhibited 35 to 183-fold reduced *in vitro* susceptibility to regdanvimab, compared with the wild-type SARS-CoV-2, but regdanvimab showed an anti-viral effect in a delta variant–infected mouse model (Ryu et al., 2021; Cox et al., 2023). Another clinical study also suggested that regdanvimab would be effective in delta variant–infected COVID-19 patients, but the number of patients in that study was small, and statistical significance was not found (Jang et al., 2023). Although regdanvimab is currently not in use due to its markedly decreased activity against omicron variants, it is still worthwhile to discern whether regdanvimab, which exhibited decreased activity but was still effective against the delta variant in *in vitro* and animal studies, was actually clinically effective.

The present large cohort study included 792 pre-delta patients from previous studies (Lee et al., 2021; Lee et al., 2022) and evaluated the effectiveness of regdanvimab treatment in patients infected with ancestral strains and the delta variant by adding 483 pre-delta and 939 delta-confirmed patients. In this study covering the cohort of 2,214 patients with mild to moderate COVID-19, we found that regdanvimab treatment had a preventive effect against disease progression in the total cohort of pre-delta and delta-confirmed subgroups, with statistically significant effectiveness for both endpoints, O_2_ requirement and progression to severe disease, but it did not show significant effectiveness in the delta-confirmed subgroup. The percentages in the pre-delta subgroup who progressed to severe disease were 3.7% in the regdanvimab group and 8.3% in the supportive care group (data not shown). The number needed to treat (NNT) to prevent severe disease (Wen et al., 2005) was 21.7 patients in the pre-delta subgroup and 32.3 patients (an increase of approximately 1.5-fold) in the delta-confirmed subgroup, in which 4.2% vs 7.3% of each treatment group experienced progression to severe disease. Although this calculation is based on a retrospective cohort, the value for the pre-delta subgroup is similar to that deduced from the phase 2/3 trial of regdanvimab (NNT for hospitalization or O_2_ therapy was 21.3 patients) (Streinu-Cercel et al., 2022). The NNTs deduced from the phase 3 trials for other FDA-approved mAb agents range from 20 to 30 patients (20.4 patients for bamlanivimab/etesevimab (Dougan et al., 2021), 30.3 patients in the 1200-mg arm of the casirivimab/imdevimab study (Weinreich et al., 2021), and 25 patients in the sotrovimab study (Gupta et al., 2022)), and the NNT of regdanvimab against the delta-variant is only slightly higher than that. Despite the effort to enroll as many patients as possible during the delta-outbreak period in this study, our analysis considered only 1,205 delta-confirmed patients, and the number of patients in the supportive care group was only 35.7% of that for the regdanvimab group, further hindering the statistical power of our cohort, because physicians eagerly prescribed regdanvimab during the delta-outbreak period. With the assumption that the same proportion of patients in each group would progress to severe disease if left untreated, our results would be statistically significant if more than 1,372 patients were enrolled (to reach a Z score above 1.96) (Mayo, 2010). Because the clinical effect of regdanvimab against the delta variant was not totally eliminated, several strategies to increase the effects of mAb agents for variants that show reduced *in vitro* susceptibility should be considered. A dose-up approach would be one potential option. For example, a double dose of tixagevimab/cilgavimab (Evusheld; AstraZeneca, Cambridge, UK) was recommended after the emergence of the omicron variant (NIH, 2022; Yang et al., 2023). Another strategy to increase the effectiveness of mAbs would be to select patients with higher risks. In this study, we identified the common risk factors for disease progression in the delta cohort. Among the various clinical variables, BMI ≥ 25 kg/m^2^, SpO2 < 97%, higher viral load, albumin < 4.0 g/dL, and CRP > 1.5 mg/dL were common risk factors for progression. However, because of the small number of delta-confirmed patients in our supportive care group, we could not further analyze the effects of regdanvimab in a higher risk cohort.

Another interesting finding of this study is that fewer patients in the delta-confirmed subgroup progressed to severe disease (5.0%), compared with the pre-delta subgroup (6.8%), although statistically not significantly different, even though the delta variant was well known to be more virulent than wild-type SARS-CoV-2 (Salleh et al., 2021; Twohig et al., 2022). This difference was also noticed when the supportive care groups were compared (7.3% of delta-confirmed and 8.3% of the pre-delta subgroups progressed to severe disease), which would additionally diminish the statistical preventive effect of regdanvimab against delta variant infection. This finding could have several possible explanations. First, during the delta-dominated outbreak period, younger patients (48 years of median age in delta confirmed group vs. 61 years in pre-delta period) with risk factors were admitted to the designated hospitals and received regdanvimab treatment. Although they had risk factors for disease progression, fewer of them would be expected to progress to severe disease if left untreated. Second, because COVID-19 vaccination started three months before the beginning of the delta-outbreak period, the prevalence of vaccination was higher among COVID-19 patients infected with the delta variant than among patients in the pre-delta period. In this study, one-third of mild COVID-19 patients infected with the delta variant had received at least one dose of a COVID-19 vaccine, and 10% of them were completely vaccinated, whereas none of the patients in the pre-delta period had received any vaccination. The actual case-fatality during the delta-dominated outbreak period (0.78%) in South Korea was actually lower than in the pre-delta period (1.16%), probably as a result of widely introduced vaccination (KDCA, 2022a). Also, the vaccination might offset the effectiveness of regdanvimab in delta confirmed patients. The effectiveness of mAbs in vaccinated patients need to be evaluated further. Lastly, although regdanvimab did not show significantly better outcome in delta confirmed patients, the possibility of effect of regdanvimab treatment cannot be excluded, as more patients received regdanvimab in delta group. The rate of O2 supplementation with facial mask was significantly lower in regdanvimab despite difference of composite outcome did not reach statistical significance.

This study has several limitations. It was designed as a retrospective cohort study and included mild to moderate COVID-19 patients before and after regdanvimab became available. Because clinician recognition of regdanvimab improved steadily, more patients received regdanvimab during the delta-outbreak period than in the pre-delta period. Also, the proportion of patients infected with the delta variant increased gradually during the delta-outbreak period. To address those limitation, we enrolled 2,214 COVID-19 patients in the total cohort and included 965 patients confirmed to have a delta-variant infection. Multivariable analyses were conducted in both the total cohort and delta-confirmed subgroup to adjust for potential confounding variables. We did not perform SARS-CoV-2 typing in the pre-delta period, so some patients in the pre-delta period might have been infected with other VOCs, such as the alpha variant. However, that effect would be small because the alpha variant spread to few people during April and May 2021, and the antiviral effect of regdanvimab against a pseudo-virus containing the N501Y mutation (major mutation of the alpha variant) was not much reduced (3.5 to 5.5-fold reduction) compared with the wild type, compared with the pseudo-virus containing the L452R/T478K/P681R mutation (major mutation of delta variant), which had a 28 to 98.3-fold reduction compared with the wild type (Ryu et al., 2021; Zhou et al., 2022; Cox et al., 2023). Lastly, we did not collect the data about the COVID-19 history of the patients. However, only 0.02% of COVID-19 patients were re-infected in the pre-delta period and 0.04% of those were re-infected in the delta period in South Korea, which was extremely low (KDCA, 2022b).

In conclusion, in a nationwide, multicenter, retrospective cohort study conducted during the pre-delta and delta outbreak periods, regdanvimab treatment effectively reduced progression to severe disease in the overall cohort, but did not show significant effectiveness in the delta-confirmed patients. Strategies to increase the effectiveness of mAb agents against variants with reduced *in vitro* susceptibility, such as dose increment, should be considered.

## Data availability statement

The original contributions presented in this study are included in the article/**Supplementary Material**. Further inquiries can be directed to the corresponding authors.

## Ethics statement

Review by the Institutional Review Board was exempted because the present investigation was conducted as part of a public health response, and minimal risk was expected to the participating patients.

## Author contributions

HK, YRJ, JYL, and J-HK have contributed equally to this work and share first authorship. SS, KTK, JYK, and KRP have contributed equally to this work and share corresponding authorship. All authors contributed to the article and approved the submitted version.
