# Supplementary material for: Effectiveness of regdanvimab treatment for SARS-CoV-2 delta variant, which exhibited decreased in vitro activity: a nationwide real-world multicenter cohort study
Source: Front Cell Infect Microbiol. 2023 May 15;13:1192512. doi: 10.3389/fcimb.2023.1192512 (PMC10225538; doi:10.3389/fcimb.2023.1192512)
Supplement: Supplementary file 1 [file DataSheet_1.pdf]

Ordinal disease severity scores

- **Score 1** (no limitation of daily activities)
- **Score 2** (limitation of daily activities but no need for supplemental O_2)
- **Score 3** (need for supplemental O_2 via nasal prong)
- **Score 4** (need for supplemental O_2 via facial mask)
- **Score 5** (need for high-flow supplemental O_2 or noninvasive mechanical ventilation)
- **Score 6** (need for invasive mechanical ventilation)
- **Score 7** (multi-organ failure or the need for extracorporeal membrane oxygenation therapy)
- **Score 8** (death)

Supplementary Table 1. Univariable and multivariable analyses for 21-day probability of O_2 requirement, total cohort

Factors for disease progression	Univariable analysis		Multivariable analysis	
	HR (95% CI)	*P* value	HR (95% CI)	*P* value
**Demographics**				
Age	1.020 (1.014–1.025)	<0.001		
Age > 60 years	1.448 (1.216–1.725)	<0.001		
Age > 70 years	1.950 (1.617–2.352)	<0.001	2.037 (1.643–2.526)	<0.001
Male gender	1.194 (1.002–1.422)	0.047	0.902 (0.748–1.086)	0.277
BMI	1.045 (1.024–1.066)	<0.001		
BMI ≥ 25 kg/m^2	1.563 (1.311–1.863)	<0.001	1.504 (1.254–1.803)	<0.001
BMI ≥ 30 kg/m^2	1.358 (1.031–1.789)	0.030		
Symptom onset to admission, days	1.197 (1.147–1.248)	<0.001	1.097 (1.048–1.148)	<0.001
Vaccination (incomplete or complete)	0.815 (0.621–1.069)	0.139		
Vaccination (complete only)	0.463 (0.267–0.802)	0.006	0.374 (0.212–0.660)	0.001
**Initial presentation**				
SpO_2, %	0.704 (0.649–0.763)	<0.001		
SpO_2 < 97%	2.006 (1.653–2.433)	<0.001	1.669 (1.371–2.033)	<0.001
Pneumonia	1.495 (1.184–1.887)	0.001	1.594 (1.246–2.039)	<0.001
**Initial laboratory tests**				
WBC count, $x10^3/\mu L$	1.016 (0.984–1.050)	0.332		
WBC count > 5500/μL	1.284 (1.069–1.543)	0.008		
Leukopenia (< 4000/μL)	0.769 (0.630–0.939)	0.010		
Neutrophil > 3500/μL	1.561 (1.310–1.859)	<0.001	1.390 (1.157–1.670)	<0.001
Lymphopenia (< 1500/μL)	1.488 (1.203–1.839)	<0.001		
Platelet count, $x10^3/\mu L$	0.991 (0.989–0.993)	<0.001		
Thrombocytopenia (< $150x10^3/\mu L$)	2.469 (2.071–2.943)	<0.001	1.830 (1.518–2.206)	<0.001
Total bilirubin, mg/dL	0.988 (0.787–1.241)	0.920		
Albumin, g/dL	0.308 (0.247–0.384)	<0.001		
Albumin < 4.0 g/dL	2.454 (1.974–3.051)	<0.001	1.376 (1.090–1.736)	0.007
AST, IU/L	1.004 (1.003–1.005)	<0.001		
ALT, IU/L	1.002 (1.001–1.003)	0.001		
BUN, mg/dL	1.004 (1.002–1.006)	<0.001		
BUN elevation (> 19 mg/dL)	1.888 (1.522–2.343)	<0.001		
Creatinine, mg/dL	1.175 (1.086–1.272)	<0.001		
Creatinine elevation (> 1.2 mg/dL)	1.930 (1.497–2.488)	<0.001	1.318 (1.007–1.725)	0.044
LDH, IU/L	1.000 (1.000–1.001)	0.355		
LDH elevation (> 400 IU/L)	1.138 (0.928–1.396)	0.215		
CRP, mg/dL	1.143 (1.126–1.161)	<0.001		
CRP elevation (> 1.5 mg/dL)	3.726 (3.102–4.475)	<0.001	2.487 (2.027–3.051)	<0.001
CRP elevation (> 5 mg/dL)	3.539 (2.898–4.322)	<0.001		
**Underlying diseases**				
Cardiovascular disease	1.088 (0.785–1.509)	0.612		

Respiratory disease	0.764 (0.464–1.257)	0.290		
Diabetes mellitus	1.448 (1.171–1.792)	0.001	1.278 (1.026–1.593)	0.029
Hypertension	1.373 (1.149–1.641)	<0.001	1.137 (0.928–1.394)	0.216
Liver disease	1.905 (0.475–7.639)	0.363		
Renal disease	2.354 (1.053–5.266)	0.037	0.756 (0.323–1.770)	0.519
Solid cancer, curative	0.739 (0.426–1.281)	0.281		
Solid cancer, metastasis	2.449 (1.267–4.734)	0.008	3.741 (1.907–7.339)	<0.001
Delta-confirmed	0.965 (0.808–1.152)	0.691		
**Regdanvimab treatment**	0.660 (0.552–0.789)	<0.001	0.677 (0.561–0.816)	<0.001

Initial Ct value was not included because only 1,911 patients (86.3%) were tested. Factors that exhibited extreme 95% CIs without statistical significance are not presented in this table.

Abbreviations: HR, hazard ratio; CI, confidence interval; BMI, body mass index; SpO_2, saturation of percutaneous oxygen; WBC, white blood cell; ALT, alanine aminotransferase; AST, aspartate aminotransferase; BMI, body mass index; BUN, blood urea nitrogen; LDH, lactate dehydrogenase; CRP, C-reactive protein

Supplementary Table 2. Univariable and multivariable analyses for 21-day probability of progression to severe disease, total cohort

Factors for disease progression	Univariable analysis		Multivariable analysis	
	HR (95% CI)	*P* value	HR (95% CI)	*P* value
**Demographics**				
Age	1.021 (1.010–1.032)	<0.001		
Age > 60 years	1.493 (1.063–2.097)	0.021		
Age > 70 years	1.663 (1.149–2.408)	0.007	1.574 (1.039–2.383)	0.032
Male gender	1.693 (1.195–2.400)	0.003	1.480 (1.027–2.135)	0.036
BMI	1.040 (1.001–1.081)	0.045		
BMI ≥ 25 kg/m^2	1.818 (1.286–2.569)	0.001	1.830 (1.283–2.610)	0.001
BMI ≥ 30 kg/m^2	1.292 (0.755–2.211)	0.350		
Symptom onset to admission, days	1.129 (1.040–1.224)	0.004	0.985 (0.898–1.081)	0.750
Vaccination (incomplete or complete)	0.550 (0.297–1.019)	0.058		
Vaccination (complete only)	0.137 (0.019–0.982)	0.048	0.180 (0.025–1.311)	0.091
**Initial presentation**				
SpO_2, %	0.556 (0.477–0.647)	<0.001		
SpO_2 < 97%	4.078 (2.904–5.727)	<0.001	3.104 (2.189–4.402)	<0.001
Pneumonia	1.479 (0.936–2.336)	0.093	1.696 (1.043–2.759)	0.033
**Initial laboratory tests**				
WBC count, x10^3/μL	1.024 (0.968–1.085)	0.406		
WBC count > 5500/μL	1.163 (0.811–1.668)	0.411		
Leukopenia (< 4000/μL)	0.936 (0.665–1.394)	0.840		
Neutrophil > 3500/μL	1.717 (1.220–2.417)	0.002		
Lymphopenia (< 1500/μL)	2.598 (1.581–4.268)	<0.001	2.182 (1.291–3.687)	0.004
Platelet count, x10^3/μL	0.989 (0.986–0.992)	<0.001		
Thrombocytopenia (< 150x10^3/μL)	2.357 (1.678–3.309)	<0.001	1.501 (1.051–2.142)	0.026
Total bilirubin, mg/dL	1.074 (0.767–1.505)	0.677		
Albumin, g/dL	0.226 (0.153–0.332)	<0.001		
Albumin < 4.0 g/dL	3.503 (2.406–5.101)	<0.001	2.134 (1.407–3.236)	<0.001
AST, IU/L	1.005 (1.003–1.007)	<0.001		
ALT, IU/L	1.003 (1.001–1.005)	0.010		
BUN, mg/dL	1.002 (0.996–1.008)	0.517		
BUN elevation (> 19 mg/dL)	2.221 (1.500–3.291)	<0.001	1.441 (0.930–2.233)	0.102
Creatinine, mg/dL	1.119 (0.936–1.338)	0.218		
Creatinine elevation (> 1.2 mg/dL)	2.025 (1.259–3.257)	0.004		
LDH, IU/L	1.003 (1.002–1.004)	<0.001		
LDH elevation (> 400 IU/L)	2.231 (1.571–3.169)	<0.001	1.189 (0.804–1.759)	0.386
CRP, mg/dL	1.143 (1.115–1.171)	<0.001		
CRP elevation (> 1.5 mg/dL)	4.392 (3.025–6.376)	<0.001		
CRP elevation (> 5 mg/dL)	5.320 (3.753–7.540)	<0.001	2.860 (1.956–4.182)	<0.001
**Underlying diseases**				
Cardiovascular disease	0.713 (0.333–1.526)	0.383		
Respiratory disease	0.356 (0.088–1.438)	0.147		
Diabetes mellitus	1.230 (0.802–1.887)	0.343		

Hypertension	1.249 (0.881–1.770)	0.212		
Liver disease	0.987 (0.138–7.059)	0.990		
Renal disease	1.370 (0.192–9.795)	0.754		
Solid cancer, curative	0.664 (0.211–2.084)	0.482		
Solid cancer, metastasis	0.845 (0.118–6.042)	0.867		
Delta-confirmed	0.735 (0.515–1.047)	0.088	1.045 (0.682–1.601)	0.839
**Regdanvimab treatment**	0.493 (0.344–0.707)	<0.001	0.489 (0.337–0.709)	<0.001

Initial Ct value was not included because only 1,911 patients (86.3%) were tested. Factors that exhibited extreme 95% CIs without statistical significance are not presented in this table.

Abbreviations: HR, hazard ratio; CI, confidence interval; BMI, body mass index; SpO_2, saturation of percutaneous oxygen; WBC, white blood cell; ALT, alanine aminotransferase; AST, aspartate aminotransferase; BMI, body mass index; BUN, blood urea nitrogen; LDH, lactate dehydrogenase; CRP, C-reactive protein

Supplementary Table 3. Comparison of the pre-delta and delta-confirmed patients, baseline characteristics

Characteristic	Pre-delta (n = 1,275)	Delta-confirmed (n = 939)	*P* value
**Demographics**			
Age, years	61 (50–71)	48 (33–61)	<0.001
Male gender	624 (48.9)	469 (49.9)	0.640
BMI	24.7 ± 3.7	25.2 ± 4.4	0.003
BMI ≥ 25 kg/m^2	547 (43.0)	448 (47.8)	0.027
BMI ≥ 30 kg/m^2	94 (7.4)	106 (11.3)	0.002
Symptom onset to admission, days	2 (1–4)	3 (1–5)	<0.001
Vaccination (incomplete or complete)	0 (0.0)	306 (32.6)	<0.001
Vaccination (complete only)	0 (0.0)	112 (11.9)	<0.001
**Initial presentation**			
Initial Ct value (NP swab, RdRp)	20.1 ± 6.0	17.9 ± 5.6	<0.001
SpO_2, %	97.4 ± 1.1	97.4 ± 1.0	0.269
SpO_2 < 97%	238 (18.7)	168 (17.9)	0.641
Pneumonia	865 (67.8)	859 (91.5)	<0.001
**Initial laboratory tests**			
WBC count, x10^3/μL	5.1 ± 1.9	4.8 ± 2.5	0.026
Lymphocyte count, x10^3/μL	1.3 ± 0.7	1.2 ± 0.5	<0.001
Platelet count, x10^3/μL	195.0 ± 70.6	182.8 ± 63.7	<0.001
Total bilirubin, mg/dL	0.6 ± 0.3	0.5 ± 0.5	0.048
Albumin, g/dL	4.4 ± 0.4	4.4 ± 0.4	0.173
AST, IU/L	33.4 ± 26.6	37.1 ± 35.9	0.006
ALT, IU/L	31.8 ± 37.1	35.3 ± 34.2	0.023
BUN, mg/dL	15.2 ± 8.3	14.2 ± 24.1	0.238
Creatinine, mg/dL	0.9 ± 0.6	0.9 ± 0.5	0.595
LDH, IU/L	319.4 ± 129.0	301.7 ± 141.5	0.003
CRP, mg/dL	1.8 ± 3.0	2.4 ± 3.0	<0.001
**Underlying diseases**			
Cardiovascular disease	100 (7.8)	56 (6.0)	0.088
Respiratory disease	58 (4.5)	31 (3.3)	0.140
Diabetes mellitus	266 (20.9)	97 (10.3)	<0.001
Hypertension	519 (40.7)	213 (22.7)	<0.001
Liver disease	3 (0.2)	2 (0.2)	0.913
Renal disease	10 (0.8)	2 (0.2)	0.070
Solid cancer, curative	38 (3.0)	34 (3.6)	0.401
Solid cancer, metastasis	18 (1.4)	1 (0.1)	0.001
Solid organ transplant	0 (0.0)	1 (0.1)	0.244

Data are expressed as the number (%) of patients, mean ± SD, or median (IQR) unless indicated otherwise.

Abbreviations: COVID-19, coronavirus disease 2019, BMI, body mass index; Ct, cycle threshold; NP,

nasopharyngeal; RdRp, RNA-dependent RNA polymerase; SpO2, saturation of percutaneous oxygen; WBC, white blood cell; AST, aspartate aminotransferase; ALT, alanine aminotransferase; BUN, blood urea nitrogen; CPK, creatine phosphokinase; LDH, lactate dehydrogenase; CRP, C-reactive protein; SD, standard deviation; IQR, interquartile range

Supplementary Table 4. Comparison of the pre-delta and delta-confirmed patients, treatment and outcomes

Variables	Pre-delta (n = 1,275)	Delta-confirmed (n = 939)	*P* value
**Regdanvimab**			
Regdanvimab treatment	403 (31.6)	692 (73.7)	<0.001
Interval from symptom onset to regdanvimab, days	3.4 ± 2.0	3.7 ± 1.9	0.004
Interval from admission to regdanvimab, days	1.5 ± 1.3	0.8 ± 1.2	<0.001
**Remdesivir, steroid, and antibiotics**			
Remdesivir treatment	136 (10.7)	143 (15.2)	0.001
Interval from admission to remdesivir, days	3.7 ± 3.0	2.2 ± 2.2	<0.001
Steroid treatment	186 (14.6)	244 (26.0)	<0.001
Interval from admission to steroid, days	4.0 ± 3.1	2.0 ± 2.4	<0.001
Antibiotic treatment	107 (8.4)	17 (1.8)	<0.001
Interval from admission to antibiotics, days	3.4 ± 3.5	2.1 ± 2.4	0.053
**Outcome measures**			
O_2 supplementation via nasal prong	297 (23.3)	207 (22.0)	0.488
Interval from admission to nasal prong, days	16.8 ± 7.7	16.8 ± 8.0	0.932
Composite outcome for progression to severe disease	87 (6.8)	47 (5.0)	0.076
Interval from admission to composite outcome, days	19.9 ± 4.1	20.1 ± 3.9	0.257
O_2 supplement via facial mask	24 (1.9)	20 (2.1)	0.680
Interval from admission to facial mask, days	6.1 ± 3.3	3.8 ± 2.2	0.007
O_2 supplement via HFNC	41 (3.2)	25 (2.7)	0.449
Interval from admission to HFNC, days	5.2 ± 3.1	3.0 ± 3.0	0.006
Referral to tertiary care center	56 (4.4)	21 (2.2)	0.006
Interval from admission to referral, days	5.7 ± 3.1	3.9 ± 2.1	0.017
**Live discharge after recovery without referral**	1215 (95.3)	918 (97.8)	0.002
Interval from admission to discharge, days	12.4 ± 11.7	9.8 ± 3.5	<0.001
**In-hospital mortality during follow-up period**	4 (0.3)	0 (0.0)	0.142

Data are expressed as the number (%) of patients, mean ± SD, or median (IQR) unless indicated otherwise.

Abbreviations: NA, not applicable; HFNC, high flow nasal cannula; SD, standard deviation; IQR, interquartile range

Supplementary Table 5. Baseline characteristics of the mild to moderate COVID-19 patients, delta-confirmed subgroup

Characteristic	Regdanvimab (n = 692)	Supportive care (n = 247)	*P* value
**Demographics**			
Age, years	49 (36-61)	43 (30-58)	<0.001
Male gender	343 (49.6)	126 (51.0)	0.696
BMI	25.3 ± 4.2	24.8 ± 4.8	0.145
BMI ≥ 25 kg/m^2	345 (49.9)	103 (41.7)	0.031
BMI ≥ 30 kg/m^2	74 (10.7)	32 (13.0)	0.325
Symptom onset to admission, days	2 (1-4)	3 (1-5)	0.002
Vaccination (incomplete or complete)	229 (33.1)	77 (31.2)	0.581
Vaccination (complete only)	81 (11.7)	31 (12.6)	0.725
**Initial presentation**			
Initial Ct value (NP swab, RdRp)	17.8 ± 5.6	18.3 ± 5.5	0.213
SpO_2, %	97.4 ± 1.0	97.3 ± 1.2	0.491
SpO_2 < 97%	113 (16.3)	55 (22.3)	0.037
Pneumonia	642 (92.8)	217 (87.9)	0.017
**Initial laboratory tests**			
WBC count, x10^3/μL	4.8 ± 1.7	5.0 ± 3.9	0.304
Lymphocyte count, x10^3/μL	1.2 ± 0.5	1.3 ± 0.5	0.008
Platelet count, x10^3/μL	183.4 ± 62.9	181.1 ± 66.1	0.629
Total bilirubin, mg/dL	0.5 ± 0.6	0.5 ± 0.3	0.954
Albumin, g/dL	4.42 ± 0.36	4.36 ± 0.36	0.020
AST, IU/L	36.2 ± 25.0	39.7 ± 56.2	0.348
ALT, IU/L	34.7 ± 31.0	37.0 ± 41.8	0.427
BUN, mg/dL	14.5 ± 27.1	13.5 ± 11.9	0.584
Creatinine, mg/dL	0.9 ± 0.3	0.9 ± 0.9	0.234
LDH, IU/L	291.2 ± 126.3	331.1 ± 174.3	0.001
CRP, mg/dL	2.6 ± 3.0	2.1 ± 3.1	0.049
**Underlying diseases**			
Cardiovascular disease	37 (5.3)	19 (7.7)	0.181
Respiratory disease	20 (2.9)	11 (4.5)	0.238
Diabetes mellitus	76 (11.0)	21 (8.5)	0.271
Hypertension	164 (23.7)	49 (19.8)	0.214
Liver disease	1 (0.1)	1 (0.4)	0.457
Renal disease	2 (0.3)	0	1.000
Solid cancer, curative	21 (3.0)	13 (5.3)	0.108
Solid cancer, metastasis	1 (0.1)	0	1.000
Solid organ transplant	1 (1.0)	0	1.000

Data are expressed as the number (%) of patients, mean ± SD, or median (IQR) unless indicated otherwise.

Abbreviations: COVID-19, coronavirus disease 2019, BMI, body mass index; Ct, cycle threshold; NP,

nasopharyngeal; RdRp, RNA-dependent RNA polymerase; SpO_2, saturation of percutaneous oxygen; WBC, white blood cell; AST, aspartate aminotransferase; ALT, alanine aminotransferase; BUN, blood urea nitrogen; CPK, creatine phosphokinase; LDH, lactate dehydrogenase; CRP, C-reactive protein; SD, standard deviation; IQR, interquartile range

Supplementary Table 6. Treatment and outcomes of the regdanvimab and supportive care groups, delta-confirmed subgroup

Variables	Regdanvimab (n = 692)	Supportive care (n = 247)	*P* value
**Regdanvimab**			
Regdanvimab treatment	692 (100)	0 (0.0)	NA
Interval from symptom onset to regdanvimab, days	3.7 ± 1.9	NA	NA
Interval from admission to regdanvimab, days	1 ± 1.2	NA	NA
**Remdesivir, steroid, and antibiotics**			
Remdesivir treatment	108 (15.6)	35 (14.2)	0.590
Interval from admission to remdesivir, days	2.4 ± 2.1	1.7 ± 2.3	0.113
Steroid treatment	145 (21.0)	99 (40.1)	<0.001
Interval from admission to steroid, days	2.3 ± 2.4	1.7 ± 2.4	0.062
Antibiotic treatment	16 (2.3)	1 (0.4)	0.055
Interval from admission to antibiotics, days	1 (0–4)	1 (NA)	0.588
**Outcome measures**			
O_2 supplementation via nasal prong	149 (21.5)	58 (23.5)	0.526
Interval from admission to nasal prong, days	2 ± 2.4	2.3 ± 2.7	0.371
Composite outcome for progression to severe disease	29 (4.2)	18 (7.3)	0.055
Interval from admission to composite outcome, days	3.2 ± 2.6	3.6 ± 2.9	0.652
O_2 supplement via facial mask	7 (1.0)	13 (5.3)	<0.001
Interval from admission to facial mask, days	3 (2–4)	4 (2–6)	0.345
O_2 supplement via HFNC	17 (2.5)	8 (3.2)	0.512
Interval from admission to HFNC, days	2 (1–6)	2 (1–7)	0.412
Referral to tertiary care center	12 (1.7)	9 (3.6)	0.081
Interval from admission to referral, days	4 (2–7)	4 (2–5)	1.000
**Live discharge after recovery without referral**	680 (98.3)	238 (96.4)	0.081
Interval from admission to discharge, days	9.9 ± 3.4	9.6 ± 3.8	0.379
**In-hospital mortality during follow-up period**	0 (0.0)	0 (0.0)	NA

Data are expressed as the number (%) of patients, mean ± SD, or median (IQR) unless indicated otherwise.

Abbreviations: NA, not applicable; HFNC, high flow nasal cannula; SD, standard deviation; IQR, interquartile range

Supplementary Table 7. Univariable and multivariable analyses for 21-day probability of O_2 requirement, delta-confirmed subgroup

Factors for disease progression	Univariable analysis		Multivariable analysis	
	HR (95% CI)	*P* value	HR (95% CI)	*P* value
**Demographics**				
Age	1.004 (0.996–1.013)	0.295		
Age > 60 years	0.663 (0.473–0.930)	0.017	0.966 (0.642–1.452)	0.867
Age > 70 years	1.143 (0.741–1.764)	0.544		
Male gender	0.997 (0.759–1.309)	0.981		
BMI	1.072 (1.045–1.100)	<0.001		
BMI ≥ 25 kg/m^2	1.777 (1.345–2.347)	<0.001		
BMI ≥ 30 kg/m^2	2.030 (1.432–2.876)	<0.001	2.301 (1.614–3.283)	<0.001
Symptom onset to admission, days	1.312 (1.227–1.403)	<0.001	1.159 (1.075–1.249)	<0.001
Vaccination (incomplete or complete)	0.800 (0.592–1.082)	0.148		
Vaccination (complete only)	0.459 (0.262–0.804)	0.007	0.602 (0.313–1.156)	0.127
**Initial presentation**				
Initial Ct value (NP swab, RdRp)	0.970 (0.945–0.996)	0.023	0.980 (0.954–1.006)	0.133
SpO_2, %	0.638 (0.562–0.723)	<0.001		
SpO_2 < 97%	2.065 (1.527–2.793)	<0.001	1.861 (1.355–2.557)	<0.001
Pneumonia	2.504 (1.235–5.077)	0.011	1.403 (0.648–3.039)	0.391
**Initial laboratory tests**				
WBC count, x10^3/μL	1.001 (0.949–1.056)	0.975		
WBC count > 5500/μL	1.115 (0.829–1.499)	0.471		
Leukopenia (< 4000/μL)	0.814 (0.606–1.094)	0.172		
Neutrophil > 3500/μL	1.638 (1.247–2.152)	<0.001		
Lymphopenia (< 1500/μL)	2.221 (1.491–3.308)	<0.001	1.709 (1.131–2.583)	0.011
Platelet count, x10^3/μL	0.990 (0.987–0.993)	<0.001		
Thrombocytopenia (< 150x10^3/μL)	2.667 (2.030–3.504)	<0.001	1.742 (1.300–2.335)	<0.001
Total bilirubin, mg/dL	1.084 (0.902–1.301)	0.389		
Albumin, g/dL	0.236 (0.160–0.348)	<0.001		
Albumin < 4.0 g/dL	2.457 (1.728–3.494)	<0.001	1.871 (1.292–2.708)	0.001
AST, IU/L	1.006 (1.004–1.007)	<0.001		
ALT, IU/L	1.008 (1.005–1.011)	<0.001		
BUN, mg/dL	1.003 (1.000–1.005)	0.021		
BUN elevation (> 19 mg/dL)	1.515 (1.004–2.285)	0.048	1.349 (0.854–2.129)	0.199
Creatinine, mg/dL	1.160 (1.016–1.325)	0.028		
Creatinine elevation (> 1.2 mg/dL)	1.201 (0.772–1.868)	0.417		
LDH, IU/L	1.001 (1.000–1.002)	0.029	0.999 (0.998–1.000)	0.008
LDH elevation (> 400 IU/L)	1.259 (0.914–1.734)	0.159		
CRP, mg/dL	1.187 (1.160–1.216)	<0.001		
CRP elevation (> 1.5 mg/dL)	4.414 (3.202–6.085)	<0.001	3.021 (2.141–4.261)	<0.001
CRP elevation (> 5 mg/dL)	4.134 (3.101–5.510)	<0.001		
**Underlying diseases**				

Cardiovascular disease	1.210 (0.716–2.047)	0.476		
Respiratory disease	0.567 (0.211–1.526)	0.261		
Diabetes mellitus	1.544 (1.049–2.274)	0.028	1.238 (0.804–1.908)	0.333
Hypertension	1.242 (0.912–1.690)	0.169		
Liver disease	2.081 (0.292–14.841)	0.465		
Solid cancer, curative	0.482 (0.179–1.297)	0.149		
Solid cancer, metastasis	4.535 (0.636–32.363)	0.132		
**Regdanvimab treatment**	0.911 (0.673–1.235)	0.549	0.963 (0.697–1.329)	0.817

Factors that exhibited extreme 95% CIs without statistical significance are not presented in this table.

Abbreviations: HR, hazard ratio; CI, confidence interval; BMI, body mass index; NP, nasopharyngeal; RdRp, RNA-dependent RNA polymerase; SpO_2, saturation of percutaneous oxygen; WBC, white blood cell; ALT, alanine aminotransferase; AST, aspartate aminotransferase; BMI, body mass index; BUN, blood urea nitrogen; LDH, lactate dehydrogenase; CRP, C-reactive protein

Supplementary Table 8. Univariable and multivariable analyses for 21-day probability of progression to severe disease, delta-confirmed subgroup

Factors for disease progression	Univariable analysis		Multivariable analysis	
	HR (95% CI)	*P* value	HR (95% CI)	*P* value
**Demographics**				
Age	1.010 (0.993–1.028)	0.236		
Age > 60 years	0.641 (0.310–1.325)	0.230		
Age > 70 years	0.614 (0.191–1.976)	0.413		
Male gender	1.047 (0.591–1.855)	0.875		
BMI	1.050 (0.992–1.111)	0.094		
BMI ≥ 25 kg/m^2	2.156 (1.179–3.942)	0.013	1.987 (1.068–3.697)	0.030
BMI ≥ 30 kg/m^2	1.388 (0.622–3.099)	0.423		
Symptom onset to admission, days	1.275 (1.108–1.466)	0.001	1.001 (0.848–1.182)	0.992
Vaccination (incomplete or complete)	0.622 (0.317–1.223)	0.169		
Vaccination (complete only)	0.157 (0.022–1.136)	0.067	0.231 (0.032–1.691)	0.149
**Initial presentation**				
Initial Ct value (NP swab, RdRp)	0.933 (0.880–0.990)	0.022	0.934 (0.880–0.992)	0.025
SpO_2, %	0.492 (0.381–0.636)	<0.001		
SpO_2 < 97%	4.693 (2.649–8.315)	<0.001	4.309 (2.411–7.701)	<0.001
Pneumonia	4.360 (0.601–31.611)	0.145		
**Initial laboratory tests**				
WBC count, x10^3/μL	1.024 (0.952–1.102)	0.518		
WBC count > 5500/μL	1.087 (0.582–2.031)	0.793		
Leukopenia (< 4000/μL)	1.055 (0.582–1.913)	0.860		
Neutrophil > 3500/μL	1.776 (0.999–3.156)	0.050		
Lymphopenia (< 1500/μL)	7.604 (1.845–31.345)	0.005	5.119 (1.233–21.259)	0.025
Platelet count, x10^3/μL	0.989 (0.983–0.995)	<0.001		
Thrombocytopenia (< 150x10^3/μL)	2.048 (1.155–3.632)	0.014	1.144 (0.633–2.069)	0.656
Total bilirubin, mg/dL	1.051 (0.665–1.660)	0.831		
Albumin, g/dL	0.112 (0.051–0.247)	<0.001		
Albumin < 4.0 g/dL	4.697 (2.543–8.673)	<0.001	3.382 (1.790–6.389)	<0.001
AST, IU/L	1.006 (1.004–1.008)	<0.001		
ALT, IU/L	1.010 (1.005–1.014)	<0.001		
BUN, mg/dL	0.999 (0.985–1.014)	0.924		
BUN elevation (> 19 mg/dL)	2.060 (0.963–4.409)	0.063	1.929 (0.873–4.261)	0.104
Creatinine, mg/dL	1.084 (0.754–1.559)	0.664		
Creatinine elevation (> 1.2 mg/dL)	1.180 (0.467–2.981)	0.727		
LDH, IU/L	1.003 (1.002–1.004)	<0.001		
LDH elevation (> 400 IU/L)	2.780 (1.553–4.979)	0.001	1.098 (0.566–2.129)	0.782
CRP, mg/dL	1.206 (1.153–1.260)	<0.001		
CRP elevation (> 1.5 mg/dL)	6.962 (3.119–15.542)	<0.001	4.169 (1.841–9.442)	0.001
CRP elevation (> 5 mg/dL)	6.885 (3.885–12.200)	<0.001		
**Underlying diseases**				
Cardiovascular disease	0.687 (0.167–2.833)	0.604		
Respiratory disease	0.640 (0.088–4.643)	0.659		

Diabetes mellitus	1.270 (0.539–2.992)	0.584		
Hypertension	0.803 (0.388–1.661)	0.554		
Liver disease	3.058 (0.422–22.172)	0.269		
Solid cancer, curative	1.156 (0.280–4.764)	0.841		
**Regdanvimab treatment**	0.568 (0.316–1.024)	0.060	0.665 (0.349–1.268)	0.216

Factors that exhibited extreme 95% CIs without statistical significance are not presented in this table.

Abbreviations: HR, hazard ratio; CI, confidence interval; BMI, body mass index; NP, nasopharyngeal; RdRp, RNA-dependent RNA polymerase; SpO_2, saturation of percutaneous oxygen; WBC, white blood cell; ALT, alanine aminotransferase; AST, aspartate aminotransferase; BMI, body mass index; BUN, blood urea nitrogen; LDH, lactate dehydrogenase; CRP, C-reactive protein
